# Supplementary material for: BldC Delays Entry into Development To Produce a Sustained Period of Vegetative Growth in Streptomyces venezuelae
Source: mBio. 2019 Feb 5;10(1):e02812-18. doi: 10.1128/mBio.02812-18 (PMC6428758; doi:10.1128/mBio.02812-18)
Supplement: FIG S1 [file mBio.02812-18-sf001.docx]

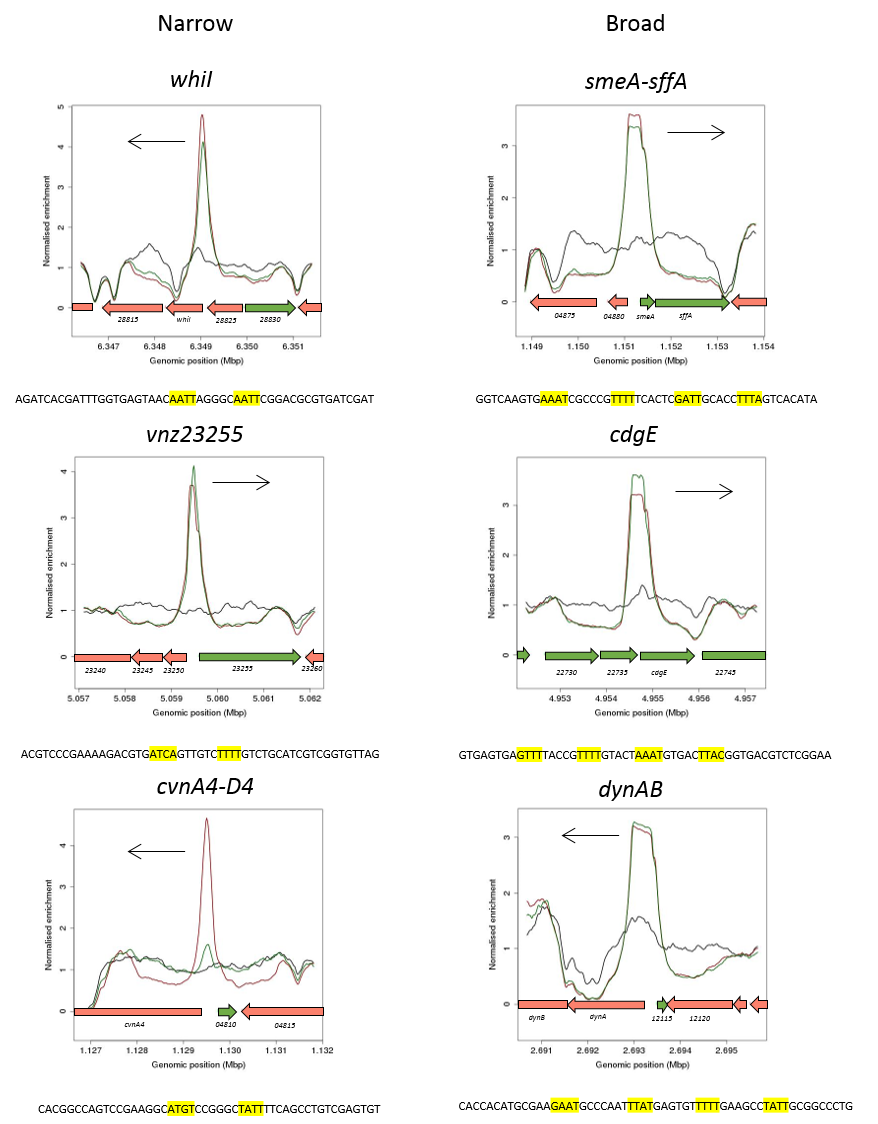


**FIG S1.** BldC ChIP-seq peaks fall into two classes. BldC binding upstream of some targets generates a broad region of enrichment (right column, labelled Broad). For *smeA*, this likely corresponds with the binding of four direct repeats by BldC, observed *in vitro* (1). Other BldC ChIP-seq targets e.g. *cdgE, dynAB* display similarly broad regions of enrichment and examination of the nucleotide sequence in these regions likewise reveals four similar and appropriately spaced direct repeats. At other BldC ChIP-seq targets, much narrower regions of enrichment are observed (left column, labelled Narrow). For *whiI*, this likely corresponds with the binding of just two direct repeats by BldC, observed *in vitro* (1). Other BldC ChIP-seq targets e.g. *vnz23255* and *cvnA4-D4* display similarly narrow regions of enrichment and examination of the nucleotide sequence in these regions likewise reveals a pair of similar and appropriately spaced direct repeats. The ChIP-seq panels are identical to those shown in Fig. 5. The AT-rich sequences of the verified (in the case of *whiI* and *smeA*) and candidate direct repeats to which BldC binds are highlighted below in yellow in the 5’-3’ direction.

**REFERENCES**

# Schumacher MA, den Hengst CD, Bush MJ, Le TB, Tran NT, Chandra G, Zeng W, Travis B, Brennan RG, Buttner MJ. 2018. The MerR-like protein BldC binds DNA direct repeats as cooperative multimers to regulate *Streptomyces* development. Nature Commun. 9:1139.
