# Supplementary material for: BldC Delays Entry into Development To Produce a Sustained Period of Vegetative Growth in Streptomyces venezuelae
Source: mBio. 2019 Feb 5;10(1):e02812-18. doi: 10.1128/mBio.02812-18 (PMC6428758; doi:10.1128/mBio.02812-18)
Supplement: TABLE S2 [file mBio.02812-18-st002.docx]

**Strains Relevant genotype/comments Source/reference**

*S.venezuelae*

NRRL B-65442 Wild type NCBI Ref: NZ_CP018074.1

SV25 Δ*bldC::apr* This work

*Sven*-pIJ10753 Wild type with pIJ10753 integrated at the ΦBT1 attachment site (1, 2)

SV25-pIJ10753 Δ*bldC::apr* with pIJ10753 integrated at the ΦBT1 attachment site This work

*Sven­*-pIJ10618 Wild type with pIJ10618 integrated at the ΦBT1 attachment site This work

SV25-pIJ10618-pKF351 Δ*bldC::apr* with pIJ10618 integrated at the ΦBT1 attachment site and pKF351 integrated at This work

the ΦC31 attachment site

**Plasmids**

pMS82 Plasmid cloning vector for the conjugal transfer of DNA from *E. coli* to *Streptomyces* spp. (3)

Integrates site specifically at the ΦBT1 attachment site (Hyg^R^)

pIJ10770 pMS82-based expression plasmid with an extended MCS and more suitable for looking at (2)

native expression levels

pIJ10618 pIJ10770 carrying a sequnece encoding bldC driven from its own promoter This work

pIJ10753 pMS82 carrying *ftsZ-ypet* driven from the *ftsZ* promoter (2)

pKF351 pSET152 carrying *ftsZ-ypet* driven from the *ftsZ* promoter (4)

**Primers Sequence**

hrdBqRT_F TGTTCTGCGCAGCCTCAATC

hrdBqRT_R CTCTTCGCTGCGACGCTCTT

whiIqRT_F CGCCTACCGCCCGAACAAGC

whiIqRT_R GTACTCCGAGGGCCCACAGG

smeAqRT_F TGTTCGGCTTCTACCTCGCGG

smeAqRT-R TCAGAACTTGATCCCGCCCA

whiDqRT-F GGCAGATTTCTCCCGCCTTC

whiDqRT-R TGGAAGAACAGGGAGCTGTCG

hupSqRT-F GACGGGTTTCGGCTCGTTC

hupSqRT-R GGGGGACCGAGGTCTTCTTG

sigFqRT-F GCGGATTCTGATGCTGCGTTA

sigFqRT-R GGCGAGGAGCCTTGACACAT

whiHqRT-F CTGGCCGACATGGTCGAGAT

whiHRT-R CTGGATGAGCAGCGAGTGGA

bldMqRT-F CCCGCATCATCATGCTCAC

bldMqRT-R CGAGGCGTCCTTGTGCAGATA

bldCcomp_F GGCGAAGCTTGGGACCTGCGGTTCGCGA

bldCcomp_R CCCGCTCGAGGGTGTTACGGGGTACTGCG

bldCdis_F AACCGAGAAGGTTCGGTTCTCCCGAGGAGGCCGCTCATGATTCCGGGGATCCGTCGACC

bldCdis_R CGGTGTTACGGGGTACTGCGGGGTGTGAGCGCGACCTCATGTAGGCTGGAGCTGCTTC

bldCcon_F TCCATCTTTGGGCGGAACG

bldCcon_R AGCGCGATCGTTGACGAAG

**REFERENCES**

1. Bush MJ, Chandra G, Findlay KC, Buttner MJ. 2017. Multi-layered inhibition of *Streptomyces* development: BldO is a dedicated repressor of whiB. Mol. Microbiol. 104: 700-711.
2. [Schlimpert S](https://www.ncbi.nlm.nih.gov/pubmed/?term=Schlimpert%20S%5BAuthor%5D&cauthor=true&cauthor_uid=28687675), [Wasserstrom S](https://www.ncbi.nlm.nih.gov/pubmed/?term=Wasserstrom%20S%5BAuthor%5D&cauthor=true&cauthor_uid=28687675), Chandra G, [Bibb MJ](https://www.ncbi.nlm.nih.gov/pubmed/?term=Bibb%20MJ%5BAuthor%5D&cauthor=true&cauthor_uid=28687675), [Findlay KC](https://www.ncbi.nlm.nih.gov/pubmed/?term=Findlay%20KC%5BAuthor%5D&cauthor=true&cauthor_uid=28687675), [Flärdh K](https://www.ncbi.nlm.nih.gov/pubmed/?term=Fl%C3%A4rdh%20K%5BAuthor%5D&cauthor=true&cauthor_uid=28687675), Buttner MJ. 2017. Two dynamin-like proteins stabilize FtsZ rings during Streptomyces sporulation. Proc Natl Acad Sci USA. 114: E6176-E6183
3. Gregory MA, Till R, Smith MCM. 2003. Integration site for *Streptomyces* phage ΦBT1 and development of site-specific integrating vectors. J. Bacteriol. 185:5320–5323.
4. [Donczew M](https://www.ncbi.nlm.nih.gov/pubmed/?term=Donczew%20M%5BAuthor%5D&cauthor=true&cauthor_uid=27248800), [Mackiewicz P](https://www.ncbi.nlm.nih.gov/pubmed/?term=Mackiewicz%20P%5BAuthor%5D&cauthor=true&cauthor_uid=27248800), [Wróbel A](https://www.ncbi.nlm.nih.gov/pubmed/?term=Wr%C3%B3bel%20A%5BAuthor%5D&cauthor=true&cauthor_uid=27248800), [Flärdh K](https://www.ncbi.nlm.nih.gov/pubmed/?term=Fl%C3%A4rdh%20K%5BAuthor%5D&cauthor=true&cauthor_uid=27248800), [Zakrzewska-Czerwińska J](https://www.ncbi.nlm.nih.gov/pubmed/?term=Zakrzewska-Czerwi%C5%84ska%20J%5BAuthor%5D&cauthor=true&cauthor_uid=27248800), [Jakimowicz D](https://www.ncbi.nlm.nih.gov/pubmed/?term=Jakimowicz%20D%5BAuthor%5D&cauthor=true&cauthor_uid=27248800). 2016. ParA and ParB coordinate chromosome segregation with cell elongation and division during *Streptomyces* sporulation. [Open Biol.](https://www.ncbi.nlm.nih.gov/pubmed/27248800) 6(4):150263.
