# Supplementary material for: BldC Delays Entry into Development To Produce a Sustained Period of Vegetative Growth in Streptomyces venezuelae
Source: mBio. 2019 Feb 5;10(1):e02812-18. doi: 10.1128/mBio.02812-18 (PMC6428758; doi:10.1128/mBio.02812-18)
Supplement: TEXT S1 [file mBio.02812-18-s0001.docx]

**Chromatin immunoprecipitation-sequencing (ChIP-seq).** To carry out the ChIP-seq experiments, duplicate cultures of *S. venezuelae* and the congenic *bldC* null mutant strain SV25 were grown for 10 h and 14 h in MYM liquid medium. Cultures were examined by microscopy to confirm the expected developmental stage. Formaldehyde was added to cultures at a final concentration of 1% (v/v) and incubation was continued for 30 min. Glycine was then added to a final concentration of 125 mM to stop the cross-linking. Cultures were left at room temperature (RT) for 5 min before the mycelium was harvested and washed twice in PBS buffer pH 7.4. Each mycelial pellet was resuspended in 0.5 ml lysis buffer (10 mM Tris HCl pH 8.0, 50 mM NaCl) containing 10 mg/ml lysozyme and protease inhibitor (Roche Applied Science) and incubated at 37 ºC for 25 min. An equal volume of IP buffer (100 mM Tris HCl pH 8, 250 mM NaCl, 0.5% Triton-X-100, 0.1% SDS) containing protease inhibitor was added and samples were chilled on ice. Samples were sonicated for 8 cycles of 20 s each at 8 microns to shear the chromosomal DNA into fragments ranging from 300-1000 bp in size. Samples were centrifuged twice at 13,000 rpm at 4 ºC for 15 min to clear the cell extract. The supernatant was incubated with 10% (v/v) protein A-sepharose (Sigma) for 1 h on a rotating wheel to remove non-specifically binding proteins. Samples were then centrifuged for 15 min at 4ºC and 13,000 rpm to remove the beads. Supernatants were incubated with 10% (v/v) anti-BldC antibody (1) overnight at 4 ºC with rotation. Subsequently, 10% (v/v) protein A-sepharose was added to precipitate BldC and incubation was continued for 4 h. Samples were centrifuged at 3500 rpm for 5 min and the pellets were washed twice with 0.5x IP buffer, and then twice with 1x IP buffer. Each pellet was incubated overnight at 65 ºC in 150 µl IP elution buffer (50 mM Tris HCl pH 7.6, 10 mM EDTA, 1% SDS) to reverse cross-links. Samples were centrifuged at 13,000 rpm for 5 min to remove the beads. Each pellet was re-extracted with 50 µl TE buffer (10 mM Tris HCl pH 7.4, 1 mM EDTA) and the supernatant incubated with 0.2 mg/ml Proteinase K (Roche) for 2 h at 55ºC. The resulting samples were extracted with phenol-chloroform and further purified using QiaQuick columns, eluting in 50 µl EB buffer (Qiagen). Library construction and sequencing were performed by Genewiz (NJ, USA), using Illumina Hiseq (2 x 150bp configuration, trimmed to 100bp).

**RNA preparation and RNA-seq.** Mycelial pellets from duplicate MYM cultures were washed in PBS and resuspended in 900 µl lysis solution (400 µl phenol [pH4.3], 100 µl chlorophorm:isoamyl alcohol (24 : 1) and 400 µl RLT buffer [Qiagen]) with lysing matrix B (MP Biomedicals) and homogenized using a FastPrep FP120 Cell Disruptor (Thermo Savant). Two pulses of 30 s of intensity 6.0 were applied with cooling down for 1 min on ice between pulses. Supernatants were centrifuged for 15 min, full-speed on a bench-top centrifuge at 4°C and then treated according to the instructions given in the RNEasy Kit (Qiagen). The RNA samples were treated with on-column DNase I (Qiagen), followed by an additional DNase I treatment (Turbo DNA-free, Ambion) until they were free of DNA contamination (determined by PCR amplification of *hrdB*). For RNA-seq, RNA library preparation and sequencing were performed by Genewiz (NJ, USA). rRNA depletion was conducted using the Illumina Ribo-Zero rRNA removal kit and paired-end sequencing conducted by Illumina Hiseq (2x150bp configuration).

**Data analysis.** A combination of stand-alone tools, Perl scripts, BioPerl toolkit (<https://bioperl.org/>) and R (<https://www.R-project.org/>.) was used to carry out analysis of RNA-seq and ChIP-seq data sets. Where possible, GNU Parallel (<https://doi.org/10.5281/zenodo.1146014>) was used to process multiple data sets in parallel.

**RNA-seq data analysis** The reads in the fastq files received from the sequencing contractor were aligned to the *S. venezuelae* genome (GenBank accession number CP018074) using the bowtie2 (2) software (version 2.2.9), which resulted in one SAM (.sam) file for each pair of fastq files (paired-end sequencing). The featureCounts() function of the Bioconductor package Rsubread was used to count the number of reads mapping to each gene on the chromosome (3). Quasi-likelihood F test implemented in the function glmQLFTest() of the Bioconductor package edgeR was used for differential expression analysis as described in the edgeR user's guide (4).

**ChIP-seq data analysis.** The reads in the fastq files received from the sequencing contractor were aligned to the S. venezuelae genome (GenBank accession number CP018074) using the bowtie2 (2) software (version 2.2.9), which resulted in one SAM (.sam) file for each pair of fastq files (paired-end sequencing). For each sam file, the depth command of samtools (version 1.8) was used to arrive at the depth of sequencing at each nucleotide position of the S. venezuelae chromosome (<https://www.sanger.ac.uk/science/tools/samtools-bcftools-htslib>). From the sequencing depths at each nucleotide position determined in 2, a local enrichment was calculated in a moving window of 30 nucleotides moving in steps of 15 nucleotides as (the mean depth at each nucleotide position in the 30-nt window) divided by (the mean depth at each nucleotide position in a 3000-nucleotide window centered around the 30-nucleotide window).This results in an enrichment ratio value for every 15 nucleotides along the genome. The enrichment ratios thus calculated were stored in files in the bedgraph format and were used for viewing in IGB. After ensuring good correlation between the replicates (Spearman correlation coefficient > 0.85) the mean of the replicates was calculated and used in further calculations. Enrichment in the control was subtracted from the enrichment in the WT files. Significance of enrichment was calculated assuming normal distribution of the control-subtracted enrichment values and rows ordered from low to high P-values. Association of regions of enrichment with P-values below 1e-4 with genes on the chromosome was done by simply listing genes left and right of the region. Rows of lower significance with the same context of genes were removed to leave the most significant row for each combination of left, right and "within" genes. Also, genes had to be in the right orientation and within 500 nucleotides of the enriched region for association with the region. The final list of genes (Table S1A) was ultimately checked by visual inspection of depth in the region in IGB.

**qRT-PCR.** For qRT-PCR, RNA was prepared as above and quantified (Qubit, Thermo Fisher Scientific) and equal amounts (350 ng) of total RNA from each sample was converted to cDNA using SuperScript II reverse transcriptase and random primers (Invitrogen). cDNA was then used as template in qRT-PCR performed using the SensiFAST SYBR No-ROX kit (Bioline). Three technical replicates were used for each gene. Specific qPCR primers (Table S1, final concentration of 250 nM) were used to amplify the target genes *whiI, smeA, whiD, sigF,* *hupS, whiH* and *bldM* as well as the *hrdB* reference gene. To normalize for differing primer efficiency, a standard curve was constructed using chromosomal DNA. Melting curve analysis was used to confirm the production of a specific single product from each primer pair. qRT-PCR was performed using a CFX96 Touch instrument using hardshell white PCR plates (BioRad), sealed with thermostable film covers (Thermo). PCR products were detected with SYBR green fluorescent dye and amplified according to the following protocol: 95°C, 3 min, then 45 cycles at 95°C 5 sec, 62°C 10 sec and 72°C 7 sec. Melting curves were generated at 65 to 95°C with 0.5°C increments. The BioRad CFX manager software was used to calculate starting quantity (SQ) values for *smeA* and *whiI* at each time point. These values were divided by the mean SQ value derived from the *hrdB* reference at the corresponding time points, generating a value for relative expression. The resulting values were normalised against the mean relative expression of the wild type at 10 hours. Each experiment was repeated at least once using independently generated cDNAs.

**Western Blotting.** Samples of frozen mycelium, originating from 2 ml liquid MYM samples, were resuspended in 0.4 ml ice-cold sonication buffer [20 mM Tris pH 8.0, 5 mM EDTA, 1 x EDTA-free protease inhibitors (Roche)] and sonicated (5x 15 sec on/15 sec off) at 4.5 micron amplitude. Lysates were then centrifuged at 16,000 xg for 15 min at 4˚C to remove cell debris. Total protein concentration was determined using the Bradford assay (Biorad). For detection of BldC, 1 µg of total protein from each time point was loaded in triplicate into a microplate (proteinsimple #043-165) and anti-BldC antibody (1) diluted 1:200. For detection of WhiA (the internal control), 0.5 µg of total protein from each time point was loaded and anti-WhiA antibody (5) diliuted 1:100. Protein levels, originating from the wild-type strain and the *bldC* mutant negative control were then assayed using the automated Western blotting machine Wes (ProteinSimple, San Jose, CA), according to the manufacturer’s guidelines.

**Time-lapse imaging of *S. venezuelae*.** Time-lapse fluorescence microscopy was conducted essentially as described previously (6, 7, 8). Before imaging, fresh *S. venezuelae* spores for each of the strains imaged were first prepared by inoculating 30 ml cultures of MYM with 10 µl of the appropriate spore stock or 20 µl of the appropriate mycelial culture. Cells were cultured at 30 ºC and 250 rpm until fully differentiated (16-24 h for hypersporulating strains, otherwise 36-40 h). 1 ml of each culture was spun at 400 xg for 2 min to pellet mycelium, the supernatant spores were diluted 1:50 in fresh MYM, and 50 µl was transferred to the cell loading well of a prepared B04A microfluidic plate (Merck-Millipore). The remaining (non-centrifuged) culture was filter-sterilized to obtain spent MYM that was free of spores and mycelial fragments. The ONIX manifold was then sealed to the B04A plate before transferring to the environmental chamber, pre-incubated at 30 ºC. Spores were loaded onto the B04A plate, at 4 psi for 15 seconds using the ONIX microfluidic perfusion system. Fresh MYM medium was initially set to flow at 2 psi for 3 hours during germination and imaging began after the first hour. Spent MYM medium was then set to flow at 2 psi for the remainder of the experiment.

Imaging was conducted using a Zeiss Axio Observer Z1 widefield microscope equipped with a sCMOS camera (Hamamatsu Orca FLASH 4), a metal-halide lamp (HXP 120V), a hardware autofocus (Definitive Focus), a 96-well stage insert, an environmental chamber, a 100x 1.46 NA Oil DIC objective and the Zeiss 46 HE shift free (excitation 500/25 nm, emission 535/30 nm) filter set. DIC images were captured with a 150 ms exposure time, YFP images were captured with a 100 ms exposure time. Images were taken every 30 min. In all experiments, multiple x/y positions were imaged for each strain and in each experiment. Representative images were transferred to the Fiji software package (<http://fiji.sc/Fiji>), manipulated and converted into the movie files presented here, as described previously (7).

**Scanning electron microscopy.** Colonies were mounted on the surface of an aluminum stub with optimal cutting temperature compound (Agar Scientific Ltd, Essex, UK), plunged into liquid nitrogen slush at approximately -210°C to cryopreserve the material, and transferred to the cryostage of an Alto 2500 cryotransfer system (Gatan, Oxford, England) attached to a FEI NanoSEM 450 field emission gun scanning electron microscope (FEI Ltd, Eindhoven, The Netherlands). The surface frost was sublimated at -95°C for 3½  mins before the sample was sputter coated with platinum for 2 min at 10 mA at below -110°C. Finally, the sample was moved onto the cryostage in the main chamber of the microscope, held at approximately -130°C, and viewed at 3 kV.

**REFERENCES**

1. Hunt AC, Servin-Gonzalez L, Kelemen GH, Buttner MJ. 2005. The *bldC* developmental locus of *Streptomyces coelicolor* encodes a member of a family of small DNA-binding proteins related to the DNA-binding domains of the MerR family. J Bacteriol 187: 716-728.
2. Langmead B, Salzberg S. 2012. Fast gapped-read alignment with Bowtie 2. Nat Methods 9:357-359.
3. Liao Y, Smyth GK, Shi W. 2013. The Subread aligner: fast, accurate and scalable read mapping by seed-and-vote. Nucleic Acids Res, 41, e108.
4. Lun, ATL, Chen, Y, and Smyth, GK**.** 2016. It’s DE-licious: a recipe for differential expression analyses of RNA-seq experiments using quasi-likelihood methods in edgeR. Methods Mol Biol. 1418, 391–416.
5. [Bush MJ](http://www.ncbi.nlm.nih.gov/pubmed/?term=Bush%20MJ%5BAuthor%5D&cauthor=true&cauthor_uid=24065632), [Bibb MJ](http://www.ncbi.nlm.nih.gov/pubmed/?term=Bibb%20MJ%5BAuthor%5D&cauthor=true&cauthor_uid=24065632), [Chandra G](http://www.ncbi.nlm.nih.gov/pubmed/?term=Chandra%20G%5BAuthor%5D&cauthor=true&cauthor_uid=24065632), [Findlay KC](http://www.ncbi.nlm.nih.gov/pubmed/?term=Findlay%20KC%5BAuthor%5D&cauthor=true&cauthor_uid=24065632), [Buttner MJ](http://www.ncbi.nlm.nih.gov/pubmed/?term=Buttner%20MJ%5BAuthor%5D&cauthor=true&cauthor_uid=24065632). 2013. Genes required for aerial growth, cell division, and chromosome segregation are targets of WhiA before sporulation in *Streptomyces venezuelae*. mBio 4:e00684-13.
6. Bush MJ, Chandra G, Findlay KC, Buttner MJ. 2017. Multi-layered inhibition of *Streptomyces* development: BldO is a dedicated repressor of *whiB*. Mol. Microbiol. 104:700-711.
7. Schlimpert S, Flärdh K, Buttner, MJ. 2016. Fluorescence time-lapse imaging of the complete *Streptomyces* life cycle using a microfluidic device. J Vis Exp 108:e53863.
8. [Schlimpert S](https://www.ncbi.nlm.nih.gov/pubmed/?term=Schlimpert%20S%5BAuthor%5D&cauthor=true&cauthor_uid=28687675), [Wasserstrom S](https://www.ncbi.nlm.nih.gov/pubmed/?term=Wasserstrom%20S%5BAuthor%5D&cauthor=true&cauthor_uid=28687675), [Chandra G](https://www.ncbi.nlm.nih.gov/pubmed/?term=Chandra%20G%5BAuthor%5D&cauthor=true&cauthor_uid=28687675), [Bibb MJ](https://www.ncbi.nlm.nih.gov/pubmed/?term=Bibb%20MJ%5BAuthor%5D&cauthor=true&cauthor_uid=28687675), [Findlay KC](https://www.ncbi.nlm.nih.gov/pubmed/?term=Findlay%20KC%5BAuthor%5D&cauthor=true&cauthor_uid=28687675), [Flärdh K](https://www.ncbi.nlm.nih.gov/pubmed/?term=Fl%C3%A4rdh%20K%5BAuthor%5D&cauthor=true&cauthor_uid=28687675), [Buttner MJ](https://www.ncbi.nlm.nih.gov/pubmed/?term=Buttner%20MJ%5BAuthor%5D&cauthor=true&cauthor_uid=28687675). 2017. Two dynamin-like proteins stabilize FtsZ rings during *Streptomyces* sporulation. [Proc Natl Acad Sci U S A](https://www.ncbi.nlm.nih.gov/pubmed/28687675) 114:E6176-E6183.
